# Multi-omics characterization of improved cognitive functions in Parkinson’s disease patients after the combined metabolic activator treatment: a randomized, double-blinded, placebo-controlled phase II trial

**DOI:** 10.1093/braincomms/fcae478

**Published:** 2025-01-06

**Authors:** Burak Yulug, Ozlem Altay, Xiangyu Li, Lutfu Hanoglu, Seyda Cankaya, Halil A Velioglu, Simon Lam, Hong Yang, Ebru Coskun, Ezgi Idil, Zubeyir Bayraktaroglu, Rahim Nogaylar, Ahmet Ozsimsek, Serkan Yildirim, Ismail Bolat, Metin Kiliclioglu, Cemil Bayram, Nursena Yuksel, Ozlem O Tozlu, Muhammad Arif, Saeed Shoaie, Ahmet Hacimuftuoglu, Cheng Zhang, Jens Nielsen, Hasan Turkez, Jan Borén, Mathias Uhlén, Adil Mardinoglu

**Affiliations:** Department of Neurology and Neuroscience, Faculty of Medicine, Alanya Alaaddin Keykubat University, Antalya 07070, Turkey; Science for Life Laboratory, KTH—Royal Institute of Technology, Stockholm 17165, Sweden; Science for Life Laboratory, KTH—Royal Institute of Technology, Stockholm 17165, Sweden; Department of Neurology, Faculty of Medicine, Istanbul Medipol University, Istanbul 34815, Turkey; Department of Neurology and Neuroscience, Faculty of Medicine, Alanya Alaaddin Keykubat University, Antalya 07070, Turkey; Department of Women’s and Children’s Health, Karolinska Institute, Neuroimaging Lab, Stockholm 17177, Sweden; Functional Imaging and Cognitive-Affective Neuroscience Lab, Istanbul Medipol University, Istanbul 34815, Turkey; Centre for Host-Microbiome Interactions, Faculty of Dentistry, Oral & Craniofacial Sciences, King’s College London, London WC2R 2LS, UK; Science for Life Laboratory, KTH—Royal Institute of Technology, Stockholm 17165, Sweden; Department of Neurology, Faculty of Medicine, Istanbul Medipol University, Istanbul 34815, Turkey; Department of Neurology and Neuroscience, Faculty of Medicine, Alanya Alaaddin Keykubat University, Antalya 07070, Turkey; Functional Imaging and Cognitive-Affective Neuroscience Lab, Istanbul Medipol University, Istanbul 34815, Turkey; Department of Neurology and Neuroscience, Faculty of Medicine, Alanya Alaaddin Keykubat University, Antalya 07070, Turkey; Department of Neurology and Neuroscience, Faculty of Medicine, Alanya Alaaddin Keykubat University, Antalya 07070, Turkey; Department of Pathology, Faculty of Veterinary, Atatürk University, Erzurum 25240, Turkey; Department of Pathology, Faculty of Veterinary, Atatürk University, Erzurum 25240, Turkey; Department of Pathology, Faculty of Veterinary, Atatürk University, Erzurum 25240, Turkey; Department of Medical Pharmacology, Faculty of Medicine, Atatürk University, Erzurum 25240, Turkey; Department of Molecular Biology and Genetics, Faculty of Science, Erzurum Technical University, Erzurum 25050, Turkey; Department of Molecular Biology and Genetics, Faculty of Science, Erzurum Technical University, Erzurum 25050, Turkey; Science for Life Laboratory, KTH—Royal Institute of Technology, Stockholm 17165, Sweden; Centre for Host-Microbiome Interactions, Faculty of Dentistry, Oral & Craniofacial Sciences, King’s College London, London WC2R 2LS, UK; Department of Pathology, Faculty of Veterinary, Atatürk University, Erzurum 25240, Turkey; Science for Life Laboratory, KTH—Royal Institute of Technology, Stockholm 17165, Sweden; BioInnovation Institute, Copenhagen DK-2200, Denmark; Department of Medical Biology, Faculty of Medicine, Atatürk University, Erzurum 25240, Turkey; Department of Molecular and Clinical Medicine, University of Gothenburg and Sahlgrenska University Hospital, Gothenburg 41345, Sweden; Science for Life Laboratory, KTH—Royal Institute of Technology, Stockholm 17165, Sweden; Science for Life Laboratory, KTH—Royal Institute of Technology, Stockholm 17165, Sweden; Centre for Host-Microbiome Interactions, Faculty of Dentistry, Oral & Craniofacial Sciences, King’s College London, London WC2R 2LS, UK

**Keywords:** Parkinson’s disease, combined metabolic activators, systems biology, multi-omics

## Abstract

Parkinson’s disease is primarily marked by mitochondrial dysfunction and metabolic abnormalities. We recently reported that the combined metabolic activators improved the immunohistochemical parameters and behavioural functions in Parkinson’s disease and Alzheimer’s disease animal models and the cognitive functions in Alzheimer’s disease patients. These metabolic activators serve as the precursors of nicotinamide adenine dinucleotide and glutathione, and they can be used to activate mitochondrial metabolism and eventually treat mitochondrial dysfunction. Here, we designed a randomized, double-blinded, placebo-controlled phase II study in Parkinson’s disease patients with 84 days combined metabolic activator administration. A single dose of combined metabolic activator contains L-serine (12.35 g), *N*-acetyl-L-cysteine (2.55 g), nicotinamide riboside (1 g) and L-carnitine tartrate (3.73 g). Patients were administered either one dose of combined metabolic activator or a placebo daily for the initial 28 days, followed by twice-daily dosing for the next 56 days. The main goal of the study was to evaluate the clinical impact on motor functions using the Unified Parkinson’s Disease Rating Scale and to determine the safety and tolerability of combined metabolic activator. A secondary objective was to assess cognitive functions utilizing the Montreal Cognitive Assessment and to analyse brain activity through functional MRI. We also performed comprehensive plasma metabolomics and proteomics analysis for detailed characterization of Parkinson’s disease patients who participated in the study. Although no improvement in motor functions was observed, cognitive function was shown to be significantly improved (*P* < 0.0000) in Parkinson’s disease patients treated with the combined metabolic activator group over 84 days, whereas no such improvement was noted in the placebo group (*P* > 0.05). Moreover, a significant reduction (*P* = 0.001) in Montreal Cognitive Assessment scores was observed in the combined metabolic activator group, with no decline (*P* > 0.05) in the placebo group among severe Parkinson’s disease patients with lower baseline Montreal Cognitive Assessment scores. We showed that improvement in cognition was associated with critical brain network alterations based on functional MRI analysis, especially relevant to areas with cognitive functions in the brain. Finally, through a comprehensive multi-omics analysis, we elucidated the molecular mechanisms underlying cognitive improvements observed in Parkinson’s disease patients. Our results show that combined metabolic activator administration leads to enhanced cognitive function and improved metabolic health in Parkinson’s disease patients as recently shown in Alzheimer’s disease patients. The trial was registered in ClinicalTrials.gov NCT04044131 (17 July 2019, https://clinicaltrials.gov/ct2/show/NCT04044131).

## Teaser

Yulug *et al*. reported that combined metabolic activators (CMAs) improved cognitive function in Parkinson’s disease patients. Functional MRI revealed brain network changes linked to cognitive improvements. A comprehensive multi-omics analysis identified molecular mechanisms underlying these benefits, suggesting CMA’s potential in treating cognitive decline in PD.

## Graphical Abstract

**Graphical Abstract:**
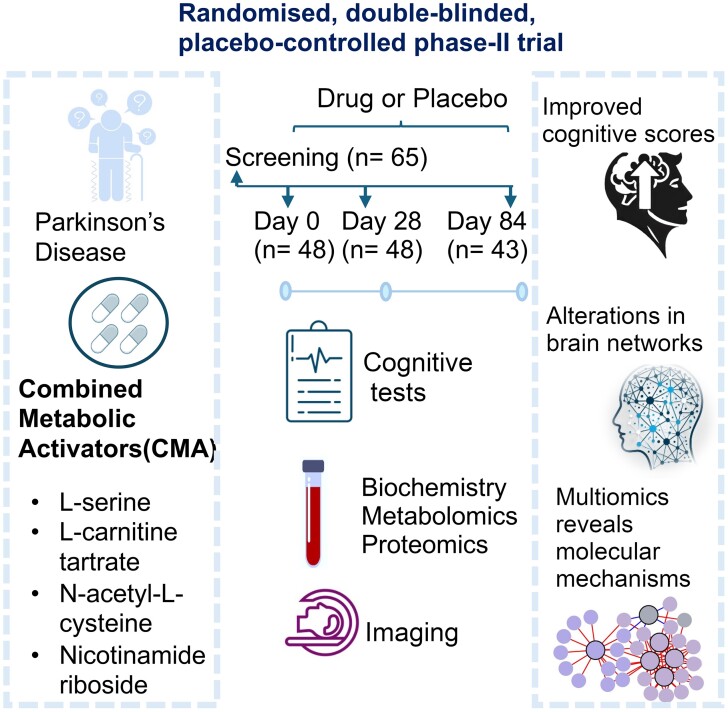

## Introduction

Parkinson’s disease is characterized by the selective degeneration of dopaminergic neurons in the substantia nigra and the presence of fibrillar aggregates, which manifests in motor and non-motor features.^1^ Although most of the Parkinson’s disease studies continue to focus on motor end-points, Parkinson’s disease is also being recognized for its complex range of non-motor symptoms,^2^ including cognitive impairment, which exists even in the prodromal stages of the disease. Indeed, growing data indicate that metabolic abnormalities associated with mitochondrial dysfunction in nerve cells might increase the risk of developing Parkinson’s disease and lead to cognitive impairment and dementia.^3^ Moreover, there is considerable evidence for the association between impaired metabolism and Parkinson’s disease development,^4-6^ consistent with a predilection to cortical anaerobic glycolysis.^5,7^ Accumulating evidence shows clinical benefits of metabolic treatments (e. g. reduced risk of Parkinson’s disease in patients with diabetes using antidiabetics),^8,9^ including some improvements in cognitive decline associated with Parkinson’s disease.^2,10^

As reported in many neurodegenerative diseases, several lines of evidence have implicated metabolic deficiency as a critical element of Parkinson’s disease pathogenesis.^10^ This is closely associated with mitochondrial dysfunction and increased oxidative stress, leading to neurodegeneration.^11,12^ Reduced mitochondrial activity and downregulation of critical genes involved in mitochondrial biogenesis have already been reported in Parkinson’s disease patients.^13^

Current Parkinson’s disease medications can only help in controlling the symptoms, and there is currently no cure for Parkinson’s disease. Hence, there is an urgent need for new therapeutic agents acting on newly defined mechanisms, such as altered brain metabolism in Parkinson’s disease. In this context, we envisaged that Parkinson’s disease patients could benefit from the treatment with combined metabolic activators (CMAs), specially designed to enhance the mitochondrial function and combat oxidative stress. This formulation includes L-carnitine tartrate, which promotes mitochondrial fatty acid uptake from the cytosol; nicotinamide riboside, an NAD^+^ precursor that boosts neuronal mitochondrial β-oxidation and facilitates fatty acid transfer across the mitochondrial membrane; and potent glutathione precursors including L-serine and *N*-acetyl-L-cysteine, which work to mitigate oxidative stress.^14-19^ Recently, we tested the effect of CMA and their individual components on the Parkinson’s disease and Alzheimer’s disease rat models and observed that CMA administration significantly alleviated hyperaemia, degeneration and necrosis in neurons.^20^ These findings were supported by decreased immunoreactivity in neurons and liver tissue of animals. CMA administration also significantly enhanced the behavioural functions in Parkinson’s disease rat animal models.^20^

Studies have shown that brain metabolism and cognitive function are closely connected, especially for complex tasks that require high amounts of metabolic energy. This can lead to brain degeneration and age-related changes associated with decreased metabolic functions in neurons. In a recent phase II study, we demonstrated that an 84-day course of oral administration of CMA produced a notable improvement in cognitive function in Alzheimer’s disease patients based on ADAS-Cog scores.^21^ The enhancement in cognitive function was associated with positive changes in cortical thickness and the preservation of hippocampal subfield volumes in the CMA group. In contrast, the placebo group showed no differences in cortical thickness but experienced a significant decline in hippocampal volume. The effectiveness of CMA administration was further supported by comprehensive plasma proteomics and metabolomics analyses, which were conducted using a multi-omics analytical platform in Alzheimer’s disease patients.

In this study, we hypothesized that the administration of CMA may improve mitochondrial function and ameliorate brain metabolism in Parkinson’s disease patients. We conducted a randomized, double-blinded, placebo-controlled phase II study to test the safety and efficacy of CMA in Parkinson’s disease patients and showed the underlying molecular mechanisms related to the improved cognitive function in Parkinson’s disease patients utilizing a comprehensive approach that includes phenomics, proteomics, metabolomics and imaging analyses.

## Materials and methods

### Human clinical trial design

Participants in this randomized, double-blinded, two-arm, parallel-group, placebo-controlled phase II trial were enrolled at the Faculty of Medicine, Istanbul Medipol University, Istanbul, Turkey, and the Faculty of Medicine, Alanya Alaaddin Keykubat University, Antalya, Turkey. Informed written consent was secured from all subjects before any trial-related activities. An independent external data-monitoring committee oversaw participant safety of conducted a risk–benefit analysis. The ethical approval was granted by the ethics committee at Istanbul Medipol University, Istanbul, Turkey (22.01.2020-07). The trial was conducted in accordance with Good Clinical Practice guidelines and adhered to the ethical principles set forth in the Declaration of Helsinki. The study is registered on ClinicalTrials.gov under Clinical Trial ID: NCT04044131 and the trial protocol is available in the Supplementary Appendix.

### Eligibility of participants

Participants were considered eligible for the study if they were over 40 years old and had mild to moderate Parkinson’s disease, classified as Stages 2–4 on the Hoehn and Yahr scale.^22^ The diagnosis of Parkinson’s disease was established using the clinical diagnostic criteria of the UK Parkinson’s Disease Society Brain Bank (UKPDSBB).^23-25^ Participants with a history of stroke, exposure to toxic substances or significant brain injury were excluded. Additionally, patients displaying symptoms suggestive of Parkinson-plus syndrome, such as pyramidal, cerebellar, autonomic dysfunctions and gaze paralysis during the neurological examination, were also excluded. For a comprehensive understanding of the inclusion, exclusion and randomization criteria for the study, please refer to the Supplementary Appendix.

### Randomization, interventions and follow-up in the clinical trial

The randomizations, interventions and follow-ups in the clinical trial protocol were performed as previously published.^21^ Participants were randomly allocated to receive either CMA or a placebo in a 2:1 ratio. An online system was applied to record patient details including data of birth, patient number and initials, while the associated randomization codes were entered into the electronic case report form. Both the clinical staff and the participants remained blinded to the treatment allocations. Treatment began on the Visit 1. Both the CMA and placebo were supplied in form of powder, with one dose contained in identical packaging. Patients were instructed to dissolve the dose in water and take one dose in the morning following breakfast and another in the evening following dinner. Each CMA dose included 12.35 g of serine, 1 g of nicotinamide riboside chloride, 2.55 g of *N*-acetylcysteine and 3.73 g of L-carnitine tartrate. All participants returned for a follow-up visit on Visit 3. Further details can be found in the trial protocol (Supplementary Appendix).

### Clinical trial outcomes

The primary goal was to evaluate the clinical differences in motor and cognitive functions between participants undergoing 84 days of treatment with CMA supplementation and those receiving a placebo. The main outcome measures included the differences in Unified Parkinson’s Disease Rating Scale (UPDRS) scores between the placebo and treatment groups among Parkinson’s disease patients. Secondary outcome measures were as follows: (i) Montreal Cognitive Assessment (MoCA) test scores; (ii) differences in CNS atrophy and resting-state network activity between the placebo and treatment groups, as observed through MRI; (iii) behavioural symptoms in Parkinson’s disease patients, assessed via the Neuropsychiatric Inventory; (iv) the tolerability and safety profile of CMA supplementation; and (v) additional efficacy parameters including metabolomic and proteomic analyses. All modifications to the protocol underwent through review and received approval from the sponsor, the institutional review board, the independent ethics committee and the relevant regulatory authorities. The sample size was determined through statistical power analysis (refer to Supplementary Appendix).

The number and nature of adverse events, including serious adverse events, and any treatment discontinuations due to CMA were recorded as critical safety end-points throughout the study duration until the conclusion of the follow-up period. Vital signs, along with baseline values and treatment status, were systematically documented on both Days 0 and 84. A comprehensive enumeration of the end-points is available in the Supplementary Appendix.

### Plasma proteomics analysis

Plasma protein levels were measured using the Olink panel (Olink Bioscience, Uppsala, Sweden). The Olink protocol, detailed in prior publications,^21^ involves treating each sample with DNA-labelled antibody pairs, known as proximity probes. When these antibody pairs bind to their respective antigens, the attached DNA tails facilitate the formation of an amplicon through proximity extension, which is subsequently quantified using high-throughput real-time PCR. For the assay, 3 μL of the probe solution was combined with 1 μL of the sample, followed by overnight incubation at 4°C. Then 96 μL of an extension solution containing the necessary enzyme and PCR reagents for the pre-amplification was added. The resulting extension products were then combined with detection reagents and primers, and subjected to qPCR analysis using the BioMark HD System (Fluidigm Corporation, South San Francisco, CA). To ensure reliability, both inter- and intra-run variations were minimized by normalizing the data with an internal control and an inter-plate control. The normalized data were reported in arbitrary units [Normalized Protein eXpression (NPX)] on a log2 scale and were linearized using the formula 2^NPX, where a higher NPX value indicates a higher protein concentration. The detection limit for each assay was set at 3 SD above the negative control (background).

### Global plasma metabolomics analysis

For the global plasma metabolomics analysis, samples were obtained on Days 0 and 84 for untargeted metabolite profiling conducted by Metabolon (Durham, NC). The metabolomics analysis protocol was performed as previously published.^21^ Samples underwent processing using an automated system (MicroLab STAR, Hamilton Company, Reno, NV), with a recovery standard added prior to extraction for quality control. Proteins were precipitated using methanol, which also helped dissociate small molecules bound to proteins. This step involved vigorous shaking for 2 min (Glen Mills GenoGrinder 2000) followed by centrifugation. The resultant extract was divided into four parts: one for analysis via ultraperformance liquid chromatography-tandem mass spectroscopy (UPLC-MS/MS) with positive ion-mode electrospray ionization, another for UPLC-MS/MS with negative ion-mode electrospray ionization, a third for gas chromatography-mass spectrometry and the fourth was retained for backup purposes.

### Functional MRI recording parameters and analysis

The functional MRI (fMRI) analysis protocol was performed as previously published.^26^ A total of 28 MRI-compatible patients were recruited for the fMRI study, including 16 in the CMA group and 12 in the placebo group. Structural and functional magnetic resonance brain imaging was conducted using the 1.5T SIGNA Explorer MRI device equipped with a 16-channel head coil (General Electric Company, USA). Functional imaging of resting-state activity was obtained once patients were positioned in the MRI device and calibration was complete. Patients were instructed to keep their eyes open and remain still during the scan. The fMRI session included 255 volumes (TR 3000 ms, TE 30 ms, FA 90; TR/TE: 3000/30 ms), with a field of view (FOV) of 256 × 256 × 156 mm (FH × AP × RL), voxel size of 4 × 4 × 4 mm, flip angle of 90 and 39 slices. The anatomical T1 image of the sagittal segment was captured with 156 slices (TR/TE: 1/3.7), a FOV of 256 × 256 × 156 mm (FH × AP × RL) and a voxel size of 1 × 1 × 1 mm. The raw data were obtained from the MRI device in DICOM format and converted to NIFTI format using the dcm2bids (ver. 2.1.4) command-line tool.^27^ Anatomical and fMRI analyses were conducted using the FMRIB FSL software tools on the Linux Mint 18.3 Sylvia operating system (ver. 6.0.3, FMRIB Software Library, Oxford, https://www.fmrib.ox.ac.uk/fsl). The FSL ‘fsl anat’ software was used to extract the brain from anatomical data. The FSL FEAT program was used to complete the preprocessing stages for all subjects collectively. The MCFLIRT tool was utilized for linear registration, aligning all functional volumes to the middle volume.^28^ A high-pass filter with a time constant of 150 s, below the 0.01–0.1 Hz range where resting-state networks are observed, was applied. The smoothing kernel FWHM was set to 5 mm. Independent component analysis (ICA) was performed using the FSL ‘melodic’ tool. Linear registration was applied for the main structural native image (T1_biascorr_brain), while nonlinear registration was used for standard space (MNI152_T1_2mm). Artefacts due to general body/head movement, respiratory and cardiovascular origins, and device-dependent slow signal fluctuations in the ICA components were manually identified based on time course, frequency content and spatial distribution criteria, as described by Salimi-Khorshidi *et al*.^29^ and Griffanti *et al*.^30^ These artefacts were removed from the functional data using the fsl_regflit command, resulting in cleaned functional images. The FSL ‘applywarp’ tool was used to realign the cleaned functional ICA components of patients to the MNI152 standard brain. These components were then used for dual regression analysis, with the general linear model (GLM) statistical parameters estimate images processed using FSL’s Randomise tool for non-parametric permutation inference.

### Statistical analysis

Linear mixed-effect models (LMER, using pymer4 package in Python 3.10)^31^ were used to fit the longitudinal clinical parameters with patient ID as the random effect to account for repeated measures and fit the interactions of visit number and treatment group in the model. Subsequently, two-way ANOVA with Tukey *post hoc* test was employed to detect differences in clinical parameters between time points and groups. Cohen’s *d* effect size was calculated using the R package ‘effsize’, with the paired parameter applied when comparing differences across various visits. Metabolite profiles with over 50% missing data across the entire set of samples were excluded from the plasma metabolomics analysis. LIMMA^32^ package in R was used to analyse metabolite changes between time points and CMA versus placebo groups. Missing values were removed in pairwise comparison. *P*-values were adjusted using the Benjamini and Hochberg method. Metabolites with a false discovery rate of 5% were considered statistically significant. Metabolomics data normalization was done to eliminate batch effects, whereby raw values for each metabolite in the experimental samples were divided by the median of those samples within each instrument batch, resulting in a median of one for each batch and metabolite. The proteomics data were presented as NPX values, representing normalized protein expression on a log2 scale. Protein profiles exhibiting more than half of the data missing across the entire sample set were excluded from the plasma proteomics analysis. LIMMA was used to identify the changes between time points and between different groups. A *P* < 0.01 was considered statistically significant. MetaboAnalyst 6.0 online software was used for the enrichment analysis of significant metabolites (https://www.metaboanalyst.ca/MetaboAnalyst/).

To determine which clinical variables predict response to CMA, patients were categorized into groups based on low and high scores for each clinical variable based on the median score at Visit 1. Patients with scores at or below the median were categorized as ‘low’, while those with scores above the median were categorized as ‘high’. Statistical significance (adj. *P* < 0.05) between visits was determined by two-way ANOVA with Tukey *post hoc* analysis. Clinical variables were deemed predictive of CMA response if statistically significant changes in MoCA scores were observed in either the low or high group within the CMA treatment group, compared to the placebo group.

For the fMRI analysis, two-way mixed ANOVA was used with study/placebo groups as the independent measure and pre- and post-treatment as repeated measures. Significant interactions were examined in the following areas: differences between pre- and post-treatment groups and time effects (pre- and post-treatment differences adjusted for groups), as previously outlined by Clarkson *et al*.^33^ Age, MoCA and UPDRS scores were also included as covariates during the GLM design matrix generation. In the dual regression process, findlab resting-state maps (sensorimotor network, visuospatial network, dorsal default mode network, ventral default network, etc. https://findlab.stanford.edu/functional_ROIs.html) were used as resting-state templates.^34^ These networks were employed to illustrate network alterations between comparison groups. Finally, Mango software [ver. 4.1 (1531)] was used to visualize the results and reveal numerical cluster values.

### Generation of multi-omics networks

A multi-omics network was constructed using Spearman correlations, with significant associations (adjusted *P* < 0.05) highlighted. The analyses were performed using the SciPy and iGraph packages in Python 3.7.

## Results

### CMA improves cognition and clinical parameters in Parkinson’s disease patients

**Figure 1:**
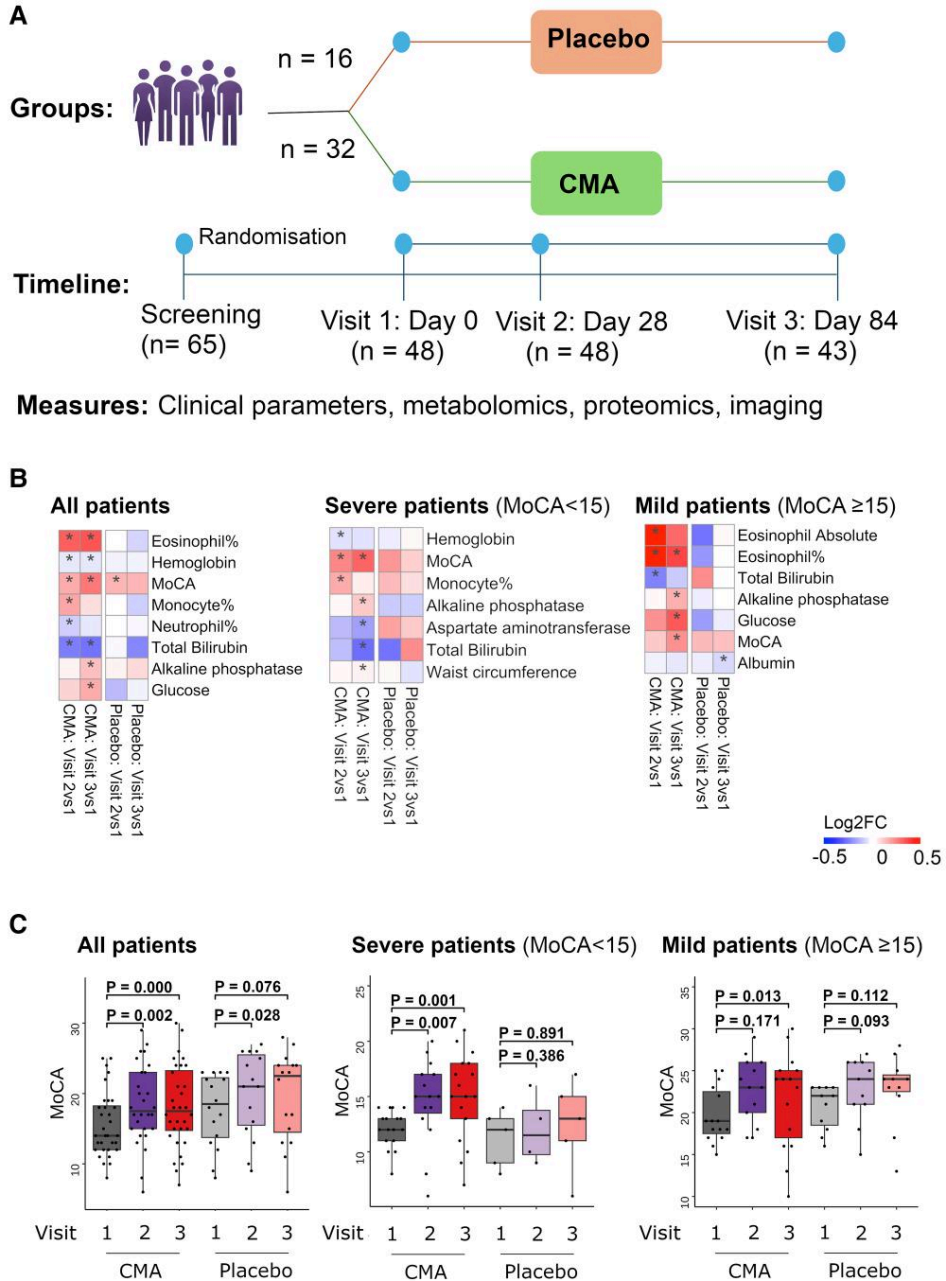
**CMA improves the MoCA scores and clinical parameters.** (**A**) Study design for testing the effects of CMA in Parkinson’s disease patients. (**B**) Differences in MoCA scores in the CMA and placebo groups on Days 0 (*n*_CMA_ = 32, *n*_placebo_ = 16), 28 (*n*_CMA_ = 32, *n*_placebo_ = 16) and 84 (*n*_CMA_ = 28, *n*_placebo_ = 15) are presented. Additionally, MoCA scores were analysed by stratifying the patients into high- and low-scored MoCA groups (≥15 MoCA score is high, *n* = 26; <15 is low, *n* = 22). (**C**) Barplots show log2FC-based alterations of the clinical variables compared to the CMA administration in both drug and placebo groups. Asterisks indicate statistical significance based on the two-way ANOVA with Tukey *post hoc* analysis (*P* < 0.05). Log2FC, log2(fold change).

We performed a randomized, double-blind, parallel, two-armed, placebo-controlled phase II study in Parkinson’s disease patients between 1 February and 31 December 2020. We screened 65 Parkinson’s disease patients and recruited 48 of the Parkinson’s disease patients in the study. These 17 participants decided not to participate in the study after learning more details about the study requirements (such as regular study visits and adhering to the treatment regimen) or for personal reasons (such as pandemic restrictions or any other personal reasons). We included patients over 40 years of age with mild and moderate Parkinson’s disease based on the Hoehn Yahr scale of 2–4. Out of the 48 patients, 32 were randomly allocated to the CMA group, while 16 were assigned to the placebo group. (Fig. 1A; Supplementary Fig. 1). Five patients withdrew from the study before Visit 3 because of the COVID-19 pandemic-related lockdown. On Days 0 (Visit 1), 28 (Visit 2) and 84 (Visit 3), we evaluated the clinical variables and analysed the differences between Visit 1 and Visit 2 as well as Visit 1 and Visit 3 in the CMA and placebo groups (Supplementary Datasets 1 and 2).

The participants had an average age of 69.7 years (41–84 years) with 83.3% being men (Supplementary Dataset 1). The mean UPDRS score was 27.44 (±12.52) for CMA and 28.06 (±12.39) for placebo on Visit 1 (Table 1; Supplementary Dataset 2). The mean MoCA score was 15.66 (±4.58) for CMA and 17.69 (±5.19) for placebo on Visit 1 (Table 1; Supplementary Dataset 2). The baseline demographic and clinical characteristics were similar between the groups (Table 1; Supplementary Dataset 1). In terms of safety, no severe adverse events were reported, and three patients experienced adverse events (Table 2).

**Table 1:** Demographics and baseline characteristics of the study population^a^

	CMA (*n* = 32)	Placebo (*n* = 16)
Age	69.88 ± 9.77	69.63 ± 9.65
Gender		
Male	26 (81.25%)	14 (87.5%)
Female	6 (18.75%)	2 (12.5%)
Ethnicity	Caucasian (100%)	Caucasian (100%)
Body mass index	29.9 ± 4.13	29.02 ± 3.7
MoCA	15.66 ± 4.58	17.69 ± 5.19
UPDRS	27.44 ± 12.52	28.06 ± 12.39

MoCA, Montreal Cognitive Assessment; UPDRS, Unified Parkinson’s Disease Rating Scale.

^a^Presented as mean ± standard deviation, except gender and ethnicity.

**Table 2:** List of adverse effects

Patient no.	Treatment	Adverse event	System organ class	Adverse event intensity	Relationship to CMA
TR10003	Active	Increased liver enzymes	Investigations	Moderate	Unknown
TR10009	Placebo	Diarrhoea	Gastrointestinal disorders	Moderate	Related
TR20039	Placebo	Peripheral oedema	General disorders	Mild	Unknown

We evaluated the clinical variables in all patients and examined the differences before and after CMA administration between the groups (Fig. 1B and C and Table 3; Supplementary Dataset 2). We found no significant changes in the primary end-point—UPDRS scores both in the drug and placebo groups at any time interval. However, we observed that mean MoCA scores (a secondary end-point) were significantly higher in the CMA group both on Visit 2 versus Visit 1 [*P* = 0.002, log2FoldChange (FC) = 0.17] and on Visit 3 versus Visit 1 (*P* = 0.000, log2FC = 0.27). We also observed significantly increased MoCA scores in the placebo group only between Visit 2 and Visit 1 (*P* = 0.028, log2FC = 0.16) whereas no significant increase between Visit 3 and Visit 1 (*P* = 0.076, log2FC = 0.15). There was no significant alteration in MoCA scores between groups at any time points. Notably, the degree of increase of MoCA was significant and it was much higher between Visit 3 and Visit 1 in the CMA group than in the placebo group, suggesting the Parkinson’s disease patients might benefit from CMA treatment after 84 days of treatment.

**Table 3:** Differences in MoCA and UPDRS scores in the CMA and placebo groups

	Placebo				CMA				CMA versus placebo					
	Log2FC		*P-*value		Log2FC		*P-*value		Log2FC			*P-*value		
	Visit 2 versus Visit 1	Visit 3 versus Visit 1	Visit 2 versus Visit 1	Visit 3 versus Visit 1	Visit 2 versus Visit 1	Visit 3 versus Visit 1	Visit 2 versus Visit 1	Visit 3 versus Visit 1	Visit 1	Visit 2	Visit 3	Visit 1	Visit 2	Visit 3
All patients														
MoCA	0.159	0.153	0.028	0.076	0.173	0.271	0.002	0.000	−0.176	−0.162	−0.058	0.233	0.219	0.564
UPDRS	−0.069	−0.129	0.767	0.597	−0.187	−0.141	0.144	0.269	−0.032	−0.151	−0.044	0.872	0.621	0.805
Severe patients														
MoCA	0.21	0.101	0.386	0.891	0.247	0.317	0.007	0.001	0.114	0.155	0.434	0.585	0.383	0.071
UPDRS	−0.229	−0.334	0.417	0.254	−0.12	−0.066	0.168	0.279	−0.218	−0.198	0.012	0.396	0.5	0.775
Mild patients														
MoCA	0.143	0.136	0.093	0.112	0.11	0.225	0.171	0.013	−0.069	−0.102	0.027	0.564	0.358	0.84
UPDRS	0.01	0.08	1	0.987	−0.044	0.02	0.705	0.811	0.079	−0.1	−0.029	0.8	0.97	0.935

MoCA, Montreal Cognitive Assessment; UPDRS, Unified Parkinson’s Disease Rating Scale.

We also examined the variations in clinical indicators by categorizing the patients into different groups of high and low MoCA-scored groups (≥15 MoCA score is high, *n* = 26; <15 is low, *n* = 22). Interestingly, we observed a significant improvement only in the low MoCA-scored (severe) patients in the CMA group both on Visit 2 (*P* = 0.007, log2FC = 0.25) and Visit 3 (*P* = 0.001, log2FC = 0.32), but no significance (*P* > 0.05) was found in the low MoCA-scored patients in the placebo group at any time points (Fig. 1B and C; Supplementary Dataset 2). Moreover, MoCA scores increased significantly in high MoCA-scored (mild) patients in the CMA (*P* = 0.013, log2FC = 0.22) group but not in the placebo (*P* = 0.112, log2FC = 0.13) at Visit 3 versus Visit 1. These results show that especially low MoCA-scored (severe) patients respond significantly to CMA administration.

Analysis of clinical variables at Visit 3 versus Visit 1 showed that serum ALP (log2FC = −0.23, *P* = 0.000), total bilirubin (log2FC = −0.27, *P* = 0.001) and glucose (log2FC = −0.17, *P* = 0.033) levels were significantly decreased only in the CMA group (Fig. 1B; Supplementary Dataset 2). We also observed that the percentage of eosinophils (*P* = 0.025, log2FC = −0.14) was significantly lower in the CMA group on Visit 3 versus Visit 1. Of note, we observed significant decreases in these inflammatory parameters on Visit 2 versus Visit 1 among patients receiving CMA (Fig. 1B; Supplementary Dataset 2).

### Response to CMA is affected by patients’ clinical profile

**Figure 2:**
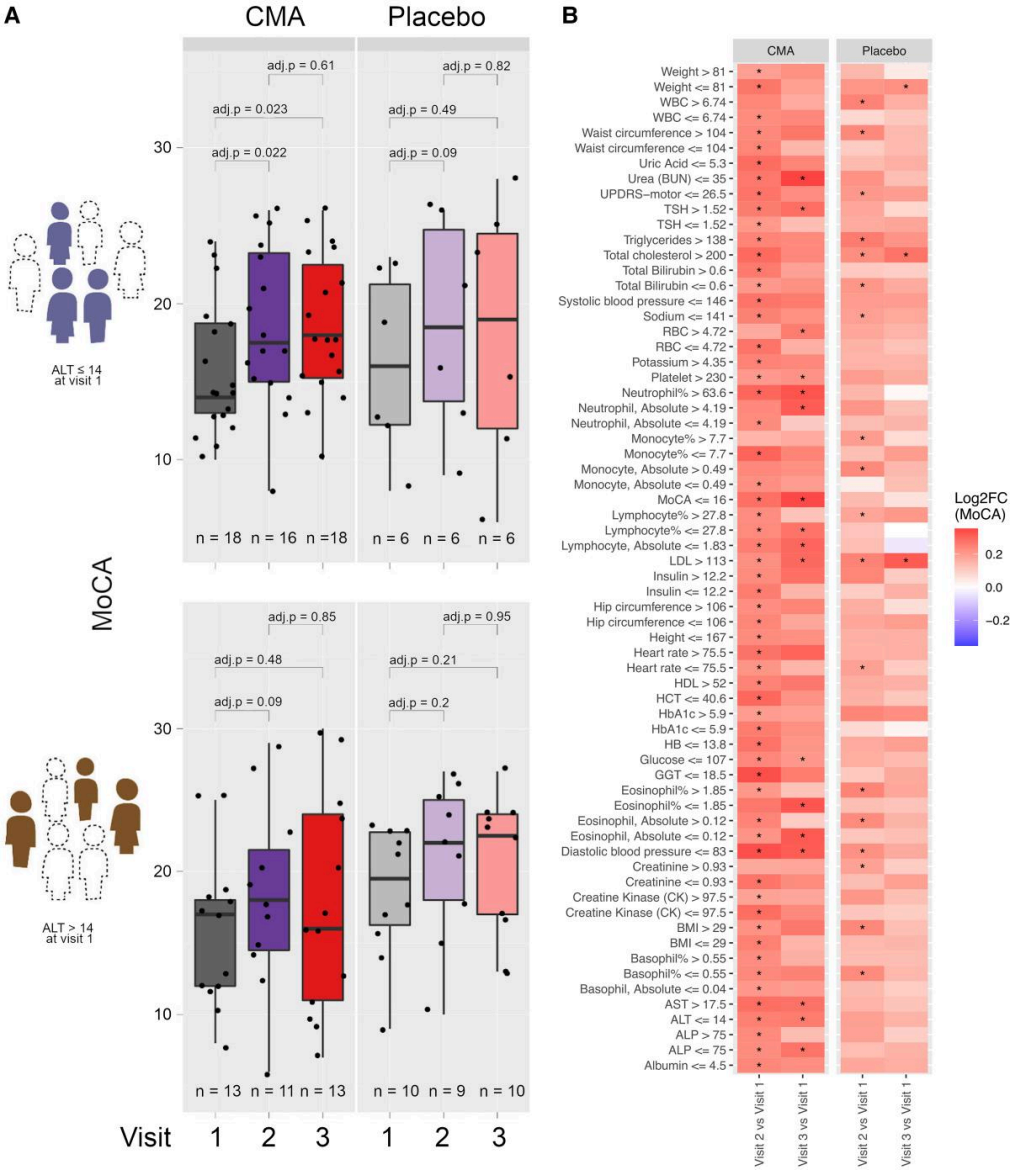
**Identification of clinical measures informative for response to CMAs.** (**A**) Distribution of the MoCA scores over visit number for patients with ALT ≤ 14 IU/L at Visit 1 (upper panel, *n* = 24) and patients with ALT > 14 IU/L at Visit 1 (lower panel, *n* = 23). (**B)** Between-visit changes to MoCA by clinical variable grouping. Only those groupings resulting in a more significant change to MoCA in CMA compared to placebo and with adj. *P* < 0.05 or better are shown. The colour scale indicates the log2FC of MoCA between visits. Statistical significance between visits was determined by two-way ANOVA with Tukey *post hoc* analysis. Asterisks indicate statistical significance. WBC, white blood cells; UPDRS motor, Unified Parkinson’s Disease Rating Scale motor; TSH, thyroid-stimulating hormone; RBC, red blood cells; LDL, low-density lipoprotein; HDL, high-density lipoprotein; HCT, haematocrit; HbA1c, haemoglobin A1c; HB, haemoglobin; GGT, gamma glutamyl transferase; BMI, body mass index; AST, aspartate aminotransferase; ALP, alkaline phosphatase.

We hypothesized that some of the patients, identified by clinical parameters, would respond to CMA better than other patients to harness the heterogeneity. We first determined whether alanine aminotransferase (ALT), a marker for liver health status, could predict response to CMA. We stratified patients receiving CMA or placebo into high- and low-ALT groups and recorded MoCA with visit day (Fig. 2A). We found that only the low-ALT group exhibited an increased MoCA score, but only when given CMA. No other group showed improvement to the same or better significance.

We then conducted a similar stratification for each clinical measure to determine other conditions in which CMA treatment leads to the best response (Fig. 2B). In addition to the aforementioned low ALT, we found low ALP, high AST, low GGT, low HCT, low glucose, low HbA1c, low uric acid, low urea, high neutrophil %, high platelet, low eosinophil count and low MoCA-scored (severe) patients could indicate a better response (adj. *P* < 0.05) to CMA. This would suggest that CMA might work better in patients with underlying clinical conditions.

### Effect of CMA on global metabolism

**Figure 3:**
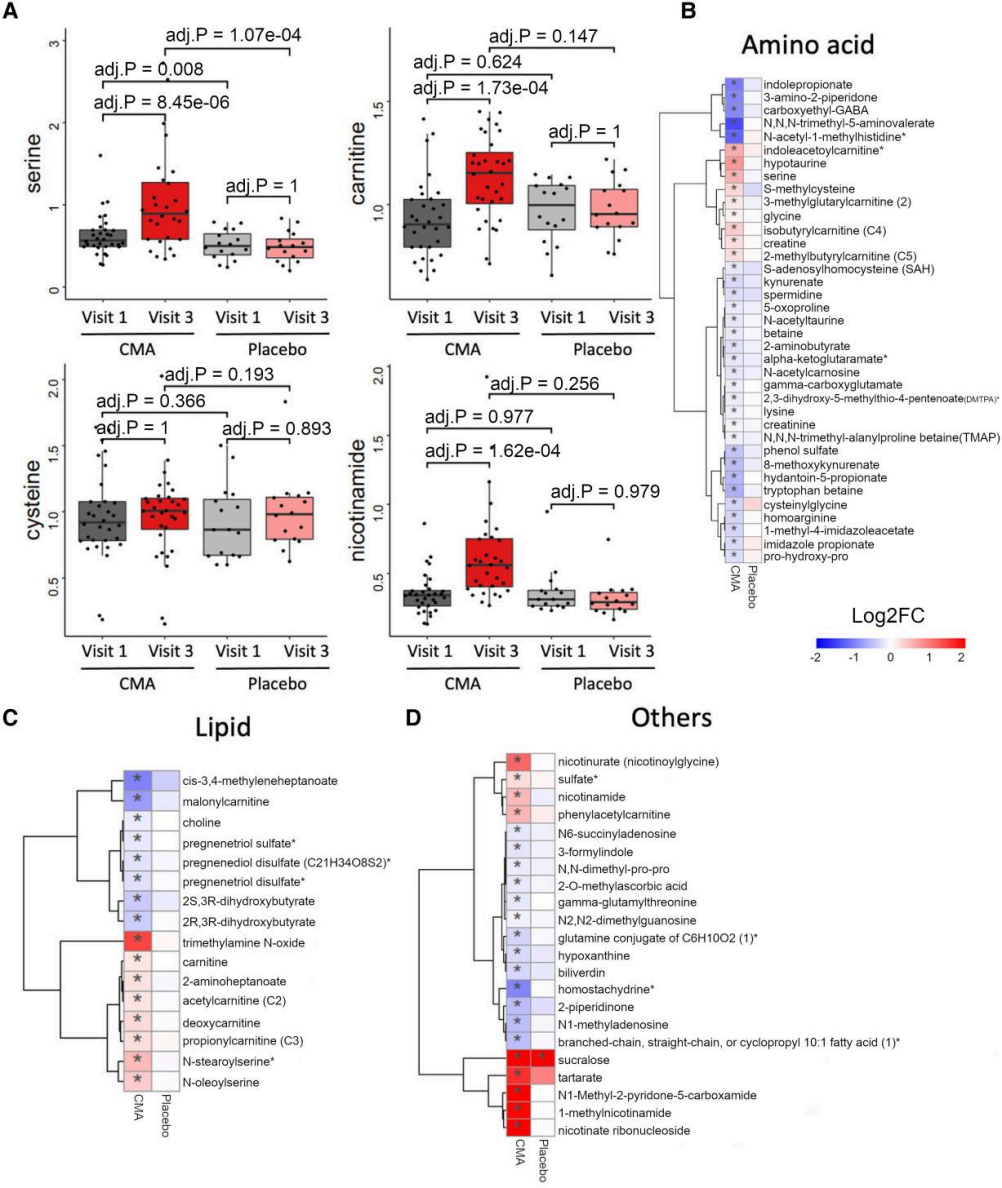
**CMA alters plasma metabolite levels.** (**A**) Differences in the plasma levels of individual CMA, including serine, carnitine, cysteine and nicotinamide, are shown in the CMA and placebo groups on Visit 1 and Visit 3. Statistical significance was determined by LIMMA, adj. *P* < 0.05. Log2FC, log2(fold change). Plasma levels of (**B**) amino acids, (**C**) lipids and (**D**) other metabolites that are significantly different between Visit 3 versus Visit 1 in the CMA (*n* = 28) and placebo (*n* = 15) groups are presented. Heatmap shows log2FC values of metabolites between Visit 3 versus Visit 1. Statistical significance was determined by LIMMA, adj. *P* < 0.05. Log2FC, log2(fold change).

To study the effect of CMA administration on global metabolism, we identified plasma metabolites whose levels were significantly (adj. *P* < 0.05) different between Visit 3 and Visit 1. We found 75 significantly different metabolites in the CMA group (Fig. 3; Supplementary Dataset 4). Assessment of plasma metabolites that showed significant differences on Visit 3 versus Visit 1 in each group showed that the metabolites related to amino acid metabolism (*n* = 37), lipid metabolism (*n* = 16) and other metabolic pathways (*n* = 22) were altered in the CMA group compared to the placebo group (Fig. 3; Supplementary Dataset 4). According to the enrichment analysis, carnitine synthesis, methionine metabolism, and nicotinate and nicotinamide metabolism were the top 3 significantly enriched pathways for significantly altered metabolites in the CMA group (Supplementary Fig. 2 and Dataset 4).

We first analysed the plasma levels of serine, carnitine, NR and cysteine and found that administration of the CMA increased the plasma serine, carnitine and nicotinamide proportionally on Visit 3 versus Visit 1 in the CMA group (Fig. 3A; Supplementary Datasets 3 and 4). In detail, plasma nicotinamide, nicotinurate, N1-methyl-2-pyridone-5-carboxamide and 1-methylnicotinamide (metabolites in NR and NAD^+^ metabolism); glycine, serine and betaine (metabolites in serine and glycine metabolism); as well as carnitine and deoxycarnitine (metabolites in carnitine metabolism) were all significantly higher on Visit 3 in the CMA group.

We found that plasma levels of creatine and glycine significantly increased on Visit 3 versus Visit 1 in the CMA group (Fig. 3B; Supplementary Dataset 4). In our clinical trial, plasma levels of *S*-adenosylhomocysteine, *N*-acetyl taurine and 2,3-dihydroxy-5-methylthio-4-pentenoate were significantly decreased on Visit 3 versus Visit 1 in the CMA group (Fig. 3B; Supplementary Dataset 4).

Higher plasma concentrations of kynurenine pathway metabolites are related to CNS disorders. We found that kynurenate, indolepropionate, 8-methoxy kynurenate and tryptophan betaine were significantly decreased on Visit 3 versus Visit 1 in the CMA group (Fig. 3B; Supplementary Dataset 4). Moreover, the plasma level of creatinine was also significantly decreased on Visit 3 versus Visit 1 in the CMA group (Fig. 3B; Supplementary Dataset 4). Additionally, our analysis revealed reduced levels of several metabolites related to histidine metabolism in the CMA group on Visit 3 versus Visit 1. Among those, *N*-acetyl-1-methylhistidine is related to decreased renal function. Furthermore, we found that plasma levels of metabolites related to the urea cycle (3-amino-2-piperidone, pro-hydroxy-pro, trimethyl-alanylproline betaine and homoarginine) and *N*,*N*,*N*-trimethyl-5-aminovalerate were significantly lower in the CMA group on Visit 3 versus Visit 1 (Fig. 3B; Supplementary Dataset 4).

Lipids are central players in the pathogenesis of neurodegenerative diseases. In our study, plasma levels of many metabolites associated with carnitine and fatty acid metabolism were significantly elevated on Visit 3 versus Visit 1 in the CMA group (Fig. 3C; Supplementary Dataset 4). Notably, plasma levels of pregnenolone steroids and dihydroxy fatty acids were significantly decreased on Visit 3 versus Visit 1 (Fig. 3C; Supplementary Dataset 4). Our comprehensive analysis also showed significantly upregulated carnitine metabolites and significantly decreased plasma bilirubin metabolites, such as biliverdin (Fig. 3D; Supplementary Dataset 4).

### Effect of CMA on plasma proteins

We measured the plasma levels of 1466 protein markers with the Olink plasma proteome profiling platform Proximity Extension Assay to quantify the plasma level of target proteins. After quality control and exclusion of proteins with missing values in >50% of samples, 1463 proteins were analysed (Supplementary Datasets 5 and 6). Proteins whose levels differed significantly between visits in the CMA and placebo groups are listed in Supplementary Dataset 6.

**Figure 4:**
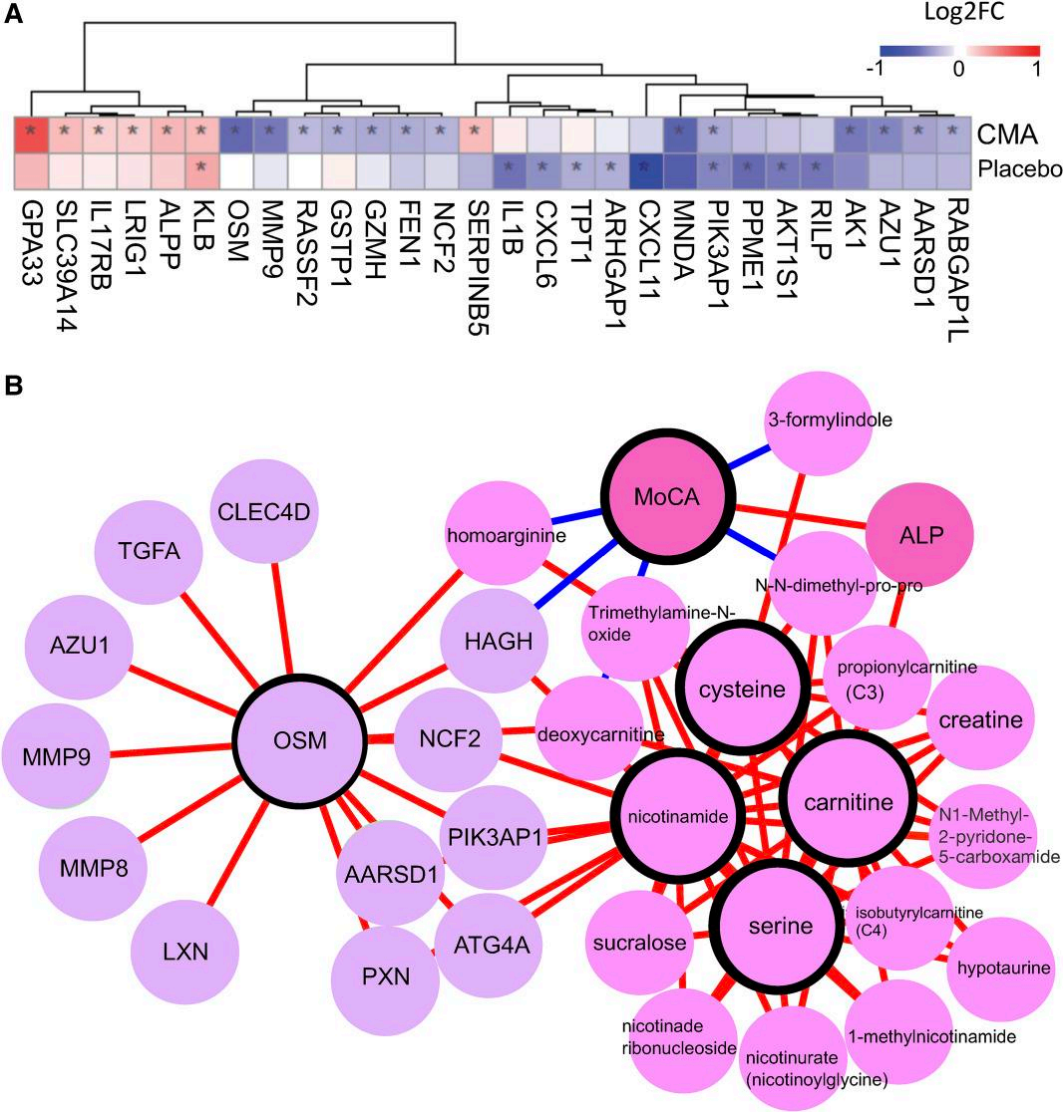
**Altered plasma protein levels and integrated multi-omics network.** (**A**) Heatmap shows log2FC-based alterations between the significantly different proteins on Visit 3 versus Visit 1 in the CMAs (*n* = 28) and placebo (*n* = 15) groups. Asterisks indicate statistical significance based on LIMMA, *P* < 0.01. (**B**) Integrated multi-omics data based on network analysis represent the neighbours of the CMA, including serine, carnitine, nicotinamide and cysteine, and the MoCA scores. Only analytes that are significantly altered in CMA on Visit 3 versus Visit 1 are highlighted. ALP, alkaline phosphatase.

We analysed the effect of CMA on the plasma protein profile and found that 20 proteins were significantly (*P* < 0.01) different in the CMA group between Visit 3 and Visit 1. Thirteen of these proteins were significantly decreased, whereas 7 were significantly increased on Visit 3 versus Visit 1. Among these proteins, we found that the plasma levels of OSM, MMP9, RASSF2, GSTP1, GZMH, FEN1, NCF2, MNDA, AK1, AZU1, AARSD1 and RAPGAP1L were significantly downregulated. The plasma levels of KLB, GPA33, SLC39A14, IL17RB, LRIG1, ALPP and SERPINB5 were significantly upregulated only in the CMA group (Fig. 4A; Supplementary Dataset 6). We observed that IL1B, CXCL6, TPT1, PIK3AP, ARHGAP1, CXCL11, PPME1, AKT1S1 and RILP were significantly (*P* < 0.01) downregulated and KLB was upregulated in the placebo group (Fig. 4A; Supplementary Dataset 6).

### Integrative multi-omics analysis

We generated a Parkinson’s disease-specific network based on multi-omics data, complemented by clinical chemistry and anthropometrics data generated in this study. The main goal of the network analysis was to elucidate the functional relationships between analytes within and between different omics data and clinical parameters. The network was generated using the same pipeline presented in the iNetModels,^35^ an interactive multi-omics network database and visualization tool, where we deposited the complete network from this study. The generated network has ∼2 million edges from 2295 nodes (40% network density, Supplementary Dataset 7).

To understand the interactions between CMA and MoCA, we extracted a subnetwork of those analytes and their top neighbours (Fig. 4B). From the subnetwork, we observed that MoCA was associated with the plasma levels of serine, trimethylamine *N*-oxide (phospholipid), deoxycarnitine, creatine and several nicotinate and nicotinamide metabolites (1-methylnicotinamide, nicotinurate, nicotinate ribonucleoside and N1-methyl-2-pyridone-5-carboxamide). MoCA was also associated with the plasma levels of several proteins, including MMP9 and OSM, as highlighted in the previous section. The plasma level of the same metabolites and proteins was significantly correlated with the CMA administration.

We also performed network centrality analysis to identify the critical nodes in the network. The top 10 most central metabolites were dominated by xenobiotics metabolites, including those associated with neurological and psychoactive drugs (lamotrigine, *O*-desmethylvenlafaxine, venlafaxine and diazepam). We also observed a nicotinamide-related metabolite (adenosine diphosphate-ribose) in that list. Meanwhile, the top 10 proteins included nervous system-related protein (NPY), a regulator of the TGF-beta process (ITGB6) and a T cell activation regulator (PRKAR1A). These results showed that integrative multi-omics network analysis could be used in elucidating the functional relationships between analytes. Our analysis also supported the results from single omics data and added new insights by discovering key analytes from the network.

### Brain network activity

**Figure 5:**
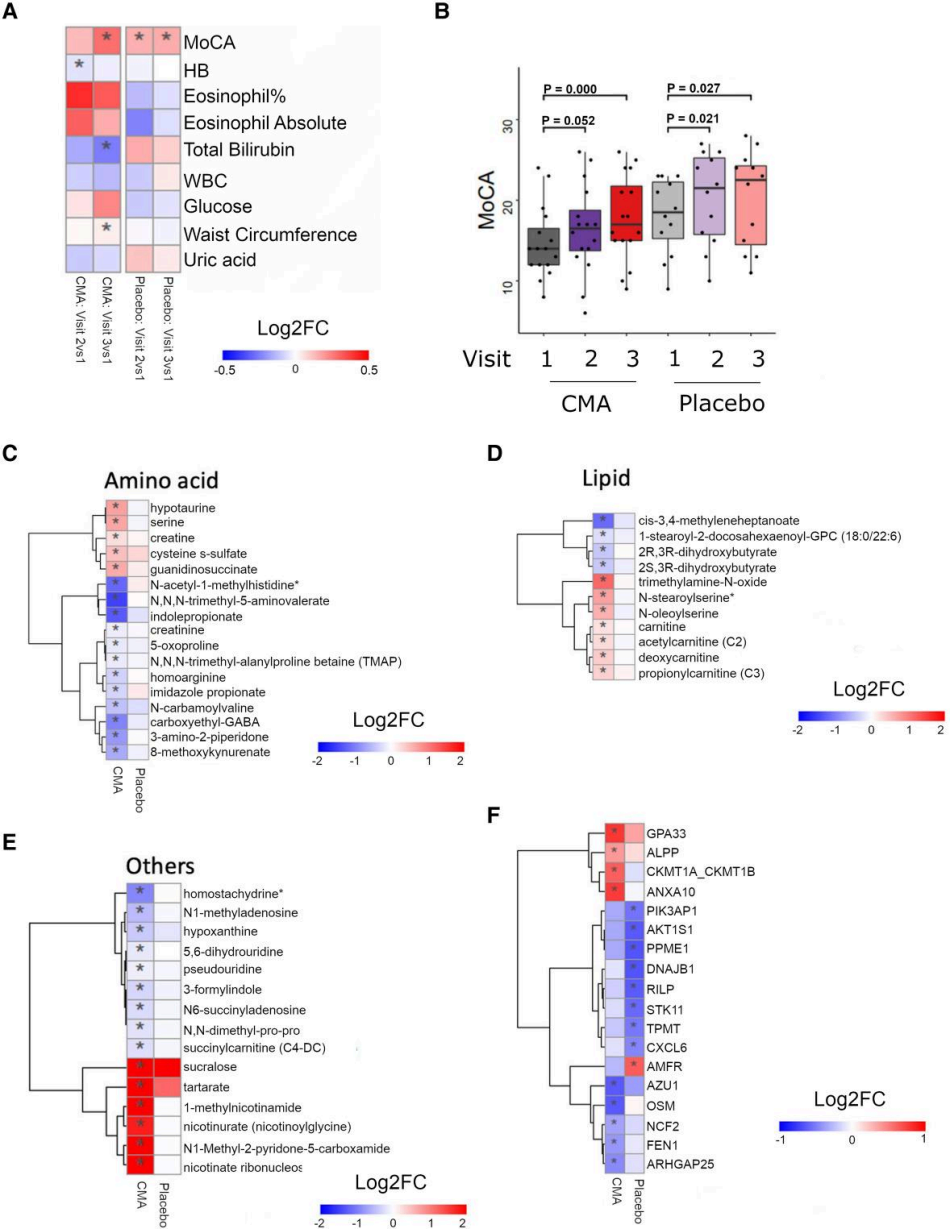
**Altered the MoCA scores, clinical parameters, plasma metabolite and protein levels in fMRI group.** (**A**) Heatmaps showing log2FC-based alterations of the clinical variables compared to the CMA administration in both CMA (*n* = 16) and placebo (*n* = 12) groups. Asterisks indicate statistical significance based on the two-way ANOVA with Tukey *post hoc* analysis, *P* < 0.05. Log2FC, log2(fold change); HB, haemoglobin; WBC, white blood cells. (**B**) Differences in MoCA scores in the CMA and placebo groups on Days 0, 28 and 84 are presented. Plasma levels of (**C**) amino acids, (**D**) lipids and (**E**) other metabolites that are significantly different between Visit 3 versus Visit 1 in the CMA and placebo groups are presented, adj. *P* < 0.05. Heatmap showing log2FC values of metabolites between Visit 3 versus Visit 1. Asterisks indicate statistical significance based on LIMMA, adj. *P* < 0.05. Log2FC, log2(fold change). (**F**) Heatmap shows log2FC-based alterations between the significantly different proteins on Visit 3 versus Visit 1 in the CMA and placebo groups. Asterisks indicate statistical significance based on LIMMA, *P* < 0.01.

Among 28 MRI-compatible patients, 16 were in the CMA group (87.5% male, mean age 67.2), and 12 were in the placebo group (83.3% male, mean age 68.1). The majority of the improved clinical parameters in the fMRI group significantly aligned with the entire patient cohort, indicating that the fMRI group was broadly representative of the entire patient cohort in terms of the beneficial effects of CMA (Fig. 5; Supplementary Dataset 8).

**Figure 6:**
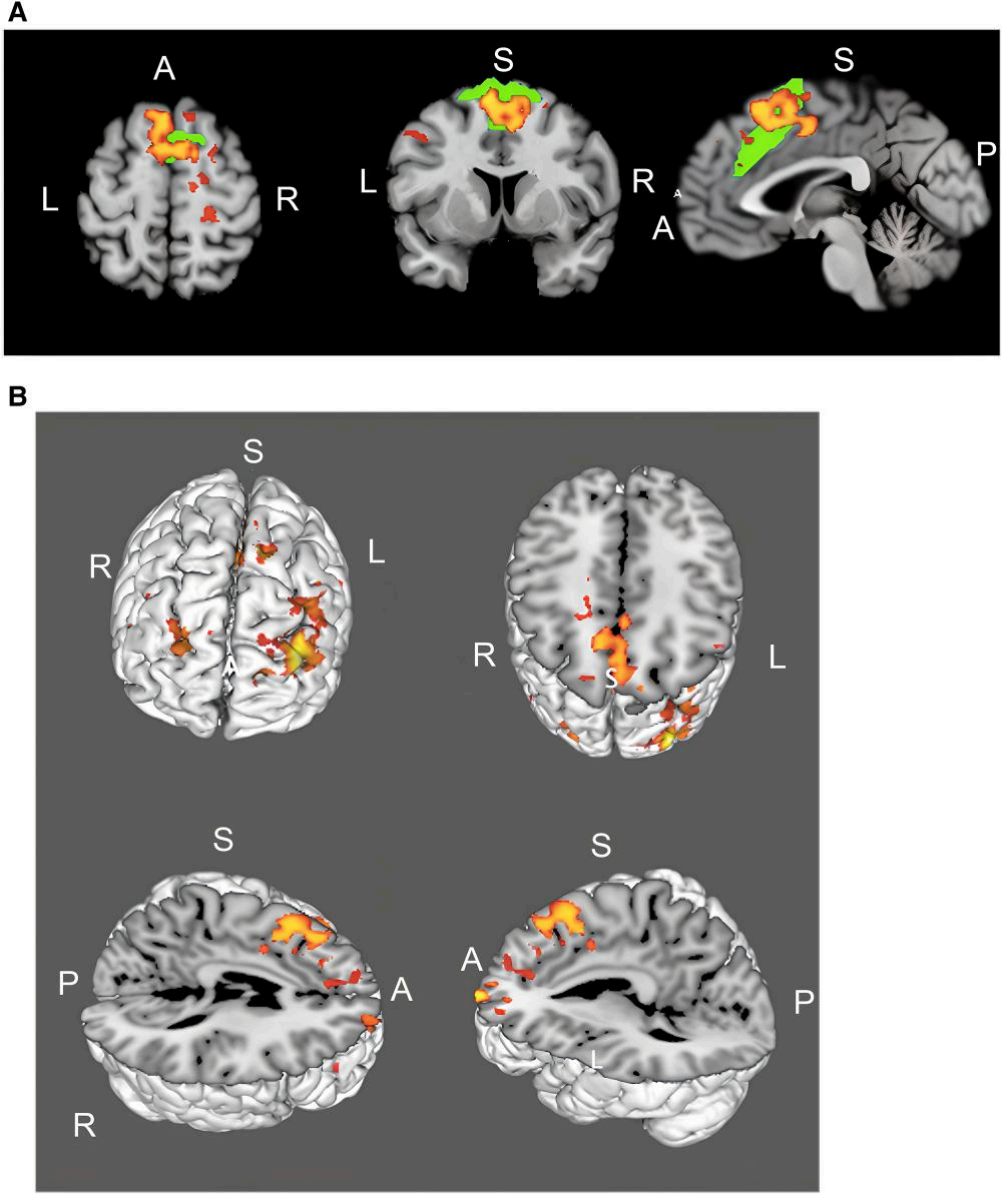
**Altered salience network activity.** (**A**) Increased salience network activity (red–yellow) in the CMA group (*P* < 0.05) based on the illustration of anterior salience networks (green) superimposed on the MNI standard brain. Two-way mixed ANOVA was used with study/placebo groups as the independent measure and pre- and post-treatment as repeated measures. Age, MoCA and UPDRS scores were also included as covariates during the GLM design matrix generation. Among 28 MRI-compatible patients, 16 were in the CMA group and 12 were in the placebo group. (**B**) 3D reconstruction of activated brain regions. L supplementary motor cortex, L paracingulate gyrus, R precentral gyrus, L–R frontal pole, L middle frontal gyrus and L–R superior frontal gyrus. S, superior; A, anterior; P, posterior; R, right; L, left.

According to the time effects (pre- and post-treatment differences adjusted for the group), there was no statistically significant increase in the resting-state network activity in the placebo group (*P* > 0.05). However, we observed a significant increase in the anterior salience network activity in the CMA group (Supplementary Dataset 9; Fig. 6, *P* < 0.05), including the bilateral frontal pole, left paracingulate gyrus, left middle frontal gyrus, left supplementary motor cortex, bilateral superior frontal gyrus and right precentral gyrus (Fig. 6).

## Discussion

In this proof-of-concept phase II study, we found that CMA administration improved cognitive functions in Parkinson’s disease patients, and it was well tolerated. Based on MoCA scores, we observed that CMA treatment significantly improved cognitive functions in Parkinson’s disease patients, especially in low MoCA-scored (severe) patients. Cognitive function in the Parkinson’s disease patients improved in the CMA group but no change in the placebo group after 84 days of CMA administration. Improvement on the Day 28 in the placebo group can be explained through a placebo effect, which could be apparent in the early stages of clinical trials associated with a reduced magnitude of change in the efficacy of outcome measures in active Parkinson’s disease treatment groups.^36^ In addition to strong associations in our cohort, we also correlated the plasma metabolite levels and functional imaging data with cognition, which is very likely a unique finding for bridging system-level biology to specific brain cognitive network functions.

Recently, we reported the results of a randomized, double-blinded, placebo-controlled human phase II clinical trial in Alzheimer’s disease patients and showed that cognitive functions in Alzheimer’s disease patients are improved by 29% in the CMA group, whereas only 14% in the placebo group after 84 days treatment,^21^ consistent with a placebo effect that is seen in the early stages of Alzheimer’s disease clinical trials.^37-39^ Moreover, a double-blinded phase I clinical study was conducted by administering only NR (one of the CMA components) in Parkinson’s disease patients.^40^ The authors have reported that the administration of NR increased cerebral NAD levels as well as other related metabolites in the CSF. Administration of NR also decreased the levels of inflammatory cytokines in serum and CSF and the study nominated NR as a potential neuroprotective therapy for Parkinson’s disease patients.^40^

We observed no significant alteration in UPDRS motor scores despite a robust cognitive response. This is, however, in line with previous studies showing that the early stages of Parkinson’s disease are likely to be more responsive to metabolic approaches than the advanced stages, in which disturbed energy metabolism cannot be easily reversed in relatively short clinical trials.^41^ Since cognitive expression has a slower metabolic deterioration rate than the pure motor pattern, indicating a probable later neurodegeneration process, the selective therapeutic response observed in this study was also unsurprising. Our improved MoCA scores, along with increased post-therapeutic alterations in lipid metabolites, also fit well with recent Parkinson’s disease studies showing an inverse correlation between lipid metabolites and MoCA scores,^42^ suggesting the key role of lipid metabolite alterations in discriminating Parkinson’s disease patients with cognitive impairment from regular Parkinson’s disease patients.

Our metabolomics data suggest several pro-energetic metabolites, particularly carnitines of the lipid–FAO pathway and nicotinamide, cysteine and serine metabolites of the TCA cycle intermediate products significantly elevated on Visit 3 versus Visit 1 in the CMA-treated group. In light of the critical signalling roles of ketone bodies in restoring disturbed energy metabolism, we also found decreased plasma levels of 2R- and 3R-hydroxybutyrate, PC and FFA metabolites indicating a possible restoration of alternative energy production pathways, activated during metabolic deficiency in Parkinson’s disease.^43-45^ CMA administration also increased levels of *N*-stearoyl serine, a lipoamino acid with numerous pleiotropic^46^ and pro-cognitive functions,^47^ also increased in the present study.

Beyond their role in energy mechanisms, lipids are involved in many critical intracellular signalling and transport processes, as the main component of cellular membranes, making them a strong candidate for cognition, even in healthy individuals.^48^ However, under neurodegenerative conditions characterized by disturbed lipid metabolism, their behaviour shifts to become more pro-inflammatory and oxidative, rather than regulatory, and contributes significantly to the acceleration of the neurodegenerative process, as in Parkinson’s disease.^49^ A good example is the detrimental role of lipids and lipid end-products in abnormal alpha-synuclein protein aggregation in the pathogenesis of Parkinson’s disease. It is worth mentioning that stearoyl-CoA desaturase, a rate-limiting enzyme in the biosynthesis of monounsaturated fatty acids, has been suggested as a player mediating lipid metabolism with synuclein aggregation.^47^

Similar beneficial alterations were also observed with increased NAD^+^, serine and cysteine levels, well-known regulators of bioenergetic imbalance and oxidative stress,^14,15,19^ resulting in the alleviation of Parkinson’s disease symptoms through increased L-dopa availability.^50-53^ The same also applies to acetyl-L-carnitine, which induces cerebral energy metabolism and cholinergic neurotransmission,^54^ which may have contributed to improved cognitive functions in our Parkinson’s disease patients, as suggested by the alterations in their plasma levels of synaptogenesis.^55^ In brief, our findings about improved lipids and TCA cycle-related metabolites are of critical significance, as altered lipid and energy metabolism and cognitive dysfunction are known to occur during Parkinson’s disease and other neurodegenerative processes.

In addition to improved lipid and TCA metabolism, we also observed significantly altered purine metabolites and creatine levels after CMA treatment, which was also shown to exert several pro-cognitive and pro-energetic effects in previous research.^56-61^ The decreased purine metabolite levels, for instance, in this study, may be interpreted as indicating increased CNS bioavailability of hypoxanthine considering its high blood–brain barrier permeability and adenosine triphosphate (ATP)-enhancing role under energy crisis conditions.^56^ Similarly, the increased creatine levels in this study accord well with the beneficial cognitive effects of creatine in healthy and Parkinson’s disease–CI patients and support the idea of its neuroprotective role as an effective mitochondrial therapy in Parkinson’s disease.^57,58^ Creatine is one of the most prevalent CNS metabolites, and reduced levels have been associated with brain tissue injury.^62^ Prior research has also shown that creatine is a central metabolite to maintain energy metabolism in the brain.^60,61^ Creatine has also been shown to induce brain oxygen utilization in Parkinson’s disease patients,^59^ suggesting its central role in maintaining energy metabolism in the brain. Deficiencies in creatine synthesis enzymes or creatine transporters can lead to intellectual disabilities and behavioural disorders. We observed that plasma levels of creatine significantly increased in the CMA group. Creatine synthesis consumes a significant portion of the body’s methyl groups, and by decreasing the need for endogenous creatine synthesis, supplementation could potentially reduce plasma homocysteine levels.^63^ Elevated plasma homocysteine levels are known to be associated with Parkinson’s disease^64^ and several *in vivo* studies have revealed the beneficial results of a low methionine diet on neurodegenerative diseases.^64^ In our clinical trial, plasma levels of *S*-adenosylhomocysteine, 2,3-dihydroxy-5-methylthio-4-pentenoate and *N*-acetyl taurine were significantly decreased in the CMA group. Additionally, we found that the plasma level of glycine, which has been extensively investigated for its positive impact on cognitive performance,^65,66^ is upregulated in the CMA group. Glycine, as part of the one-carbon metabolism, acts as a neurotransmitter in the CNS and influences cognitive functions. Increasing glycine concentrations in the brain have been shown to enhance memory and learning tasks in human studies.^67,68^

Accumulating evidence suggests an association between kidney and brain disorders, but the causal relationship between renal function and cognitive impairment remains to be established.^69^ Recent studies showed that plasma levels of *N*,*N*,*N*-trimethyl-5-aminovalerate involved in lysine metabolism indicate elevated urinary albumin excretion.^70^ Here, we found that the plasma levels of *N*,*N*,*N*-trimethyl-5-aminovalerate and creatinine were significantly decreased in the CMA group. These findings described above are consistent with our observation of decreased creatinine levels, as also suggested by recent human Parkinson’s disease data showing a direct relationship between increased serum creatinine levels and incident dementia, cognitive impairment^71^ and Parkinson’s disease progression.^72^ Furthermore, we found that plasma levels of metabolites related to the urea cycle were significantly lower in the CMA group. Consistent with the detrimental role of high urea levels on learning^73^ and Parkinson’s disease progression,^74-76^ we also observed the plasma level of 3-amino-2-piperidone, associated with urea and ornithine metabolism, decreased significantly after CMA. In addition, levels of tryptophan metabolism (i.e. kynurenate, indolepropionate, tryptophan betaine) and histidine (imidazole propionate) metabolites decreased significantly after CMA treatment, which could be associated with increased neurodegeneration and clinical cognitive impairment through increased oxidative stress and the aggregation of neurofibrillary tangles.^77,78^ Higher plasma concentrations of kynurenine pathway metabolites are related to CNS disorders.^79^ Kynurenate is the product of tryptophan metabolism and is well known for its oxidative stress-inducing effects by generating superoxide radicals and leading to cytochrome C depletion. According to previous studies, high levels of kynurenine cause cell death in natural killer cells and decrease blood pressure in the systemic inflammatory response through reactive oxygen species.^80,81^ For instance, despite its concentration-dependent dual antioxidant role, studies reported that increased bilirubin levels in Parkinson’s disease patients^82-84^ were linked with increased oxidative stress.^84^ In parallel, there is evidence of increased heme oxygenase activity of dopaminergic cells after oxidative stress,^85,86^ an enzyme responsible for the production of biliverdin.^87^ Our finding of increased hypotaurine metabolism is also consistent with the literature.^88^ For instance, a recent study identified increased hypotaurine metabolism as a compensatory neuroprotective pathway in a mouse model with alpha-synuclein.^89^

Alterations in post-treatment proteomic levels are in line with our metabolomic results suggesting the beneficial effects of CMA on brain energy metabolisms,^90^ synaptic pathology,^91^ neuroinflammation,^92-95^ oxidative injury,^96^ mitochondrial detoxification,^97,98^ intestinal integrity^99^ and cognition. Several experimental studies suggest that the altered proteins found in our study are involved in inflammation, membrane transport, DNA repair, membrane trafficking, synaptogenesis, oxidative injury and protein aggregation. For instance, ALPP and LRIG1 are well-known molecules for their antioxidant and neurotrophic properties among the increased proteins. Similarly, SLC39A14 functions as a pivotal manganese transporter in vertebrates^100^ and its deficiency is associated with rapidly progressive childhood-onset parkinsonism–dystonia due to excessive accumulation of manganese in the brain. Also, GPA33, a protein strictly limited to the intestine and responsible for intestinal integrity with unknown central functions,^99^ was increased, suggesting the role of a gut–brain axis component in neurodegenerative disorders.

Similar relevant alterations were also observed for decreased protein levels. For instance, AK1, an ATP regulator protein, has been defined in post-mortem Parkinson’s disease brains as upregulated, indicating energy dysregulation in Parkinson’s disease,^101^ which is especially relevant considering the increasing evidence of a strong link between protein aggregation, inflammation and energy deficiency in Parkinson’s disease. We consistently found significantly reduced plasma levels of MMP9, RASSF2, GSTP1, GNZMH, NCF-2, AARSD1, MNDA, OSM and FEN1, which are proteins involved in neuroinflammation, apoptosis, oxidative stress, and central and peripheral immunologic responses. Other notable observations include the downregulated levels of OSM, MNDA, AZU1 and MMP9, which are well-known neuroinflammatory markers, proven in several Parkinson’s disease models. Similar beneficial alterations have also been observed for some other proteins such as RABGAP1L and single tRNA synthetase editing domain, involved directly or indirectly in misfolded protein aggregation. We observed changes in some other molecules that are also consistent with human data, showing either a significant improving effect on the neurodegenerative process or being involved in the pathogenetic process. For instance, AZU1 and MMP-9, both important cascades of a multifunctional neuroinflammatory process and blood–brain barrier breakdown, as mentioned above, were elevated in individuals with Parkinson’s disease.^92^ Also, dysregulated levels of RABGAP1L, involved in cellular membrane trafficking, and GSTP1, a well-known molecule with attenuating functions on oxidative and endoplasmic reticulum stress,^102,103^ have been found in human dopaminergic neurons and the synaptosomal fraction of patients with Parkinson’s disease.^104^ In addition, LRIG1 was increased in the present study and was recently shown to be located in the soma and extends out into the apical dendrites of hippocampal pyramidal neurons, controlling brain-derived neurotrophic factor signalling,^105^ which is a neuroprotective and pro-cognitive molecule. Hence, our analysis suggested that CMA administration improved the plasma level of proteins associated with neurological functions in Parkinson’s disease patients.

Overall, these metabolomic and proteomic results indicate that the administration of CMA affected the global metabolism of Parkinson’s disease patients. At the same time, cognition, possibly one of the most energy-expensive functions of the brain, was most likely and positively affected after metabolic stimulation. These findings are interesting given the specific correlations of MoCA scores with specific energetic metabolites found in our study. Interestingly, these beneficial alterations in cognitive scores were also reflected in energetic cognitive networks, which play a particular role in Parkinson’s disease-related cognitive impairment. Cognitive networks with higher integrative functional dynamics, such as salience networks, may be the most sensitive part to degradation in aging and neurodegenerative diseases, which are characterized by neuronal metabolic dysfunction.^41,106^ In light of these findings, our observation of specifically increased salience network activity after metabolic stimulation was unsurprising. Consistent with the recently defined role of salience network activity in Parkinson’s disease-related cognitive impairment, we observed significantly increased metabolic activity in the salience network associated with improved cognitive functions in our Parkinson’s disease patients. Considering the role of the striatum in Parkinson’s disease-related cognitive processes, as well as the role of dopamine as an ‘anticipatory neurotransmitter molecule’ related to therapeutic expectations, especially in Parkinson’s disease placebo groups,^38,107^ it was not surprising to see a cognitive improvement in our placebo group, albeit a minimal one. In that same context, based on previous placebo neuroimaging studies, we also expected increased striatal network activity in the placebo group.^108^ However, since local metabolic contributions to placebo/sham from the striatum are weaker than the other network regions,^109^ it was entirely reasonable that we detected no striatal cognitive network activity in the placebo group. However, we determined a significant increase in salience network activity in the active Parkinson’s disease group, suggesting a real drug effect reflected in ‘easily detected’ major cognitive brain networks. In contrast, no significant network activity was observed in the placebo group, agreeing with previous observations linking MoCA scores with salience network activity in Parkinson’s disease patients.^110^

The study also has limitations to be addressed. One potential limitation is that the sample size becomes small after classifying the patients into low- and high-scored groups based on the MoCA values. Therefore, a phase III clinical trial with a larger sample size to delineate the effects of CMA on functional and structural brain alterations would be more informative. Another limitation is the placebo effect, a well-documented phenomenon, particularly in studies involving neurological outcomes, which could be attributed to several factors in our study. First, the act of participation in a clinical trial often results in increased attention and care from healthcare providers, which can enhance participants’ subjective perception of improvement. This attention effect, coupled with the participants’ expectations of benefit, might have contributed to the observed transient cognitive improvements in the placebo group which has been highlighted in several neurodegenerative studies.^37,107,111^ It is also worth considering the natural variability in MoCA scores, which can be influenced by factors such as learning effects from repeated testing, variability in test administration and day-to-day fluctuations in cognitive performance.

Therapeutic options for Parkinson’s disease patients are limited to cholinesterase inhibitors,^112^ which may be transient to replace the impaired cholinergic transmission.^113^ Also, it is still under debate whether dopamine replacement medications have cognitive side effects^114^ while improving motor symptoms in cognitively impaired Parkinson’s disease patients. To this end, there is still no available drug to revert Parkinson’s disease cognitive deficits, and, unfortunately, current treatment approaches for motor impairment are not devoid of cognitive side effects. Considering all these findings, it is crucial to better delineate the neuropathology underlying cognitive symptoms through clinical and preclinical studies. In conclusion, CMA significantly improved cognition and clinical and metabolic markers in Parkinson’s disease patients after 84 days of treatment. These findings suggest that targeting multiple pathways using CMA is a potentially effective therapeutic strategy for Parkinson’s disease patients.

## Supplementary Material

fcae478_Supplementary_Data

## Data Availability

The data supporting the findings of this study are available in the Supplementary material. Raw data can be obtained from the corresponding author upon reasonable request. The codes are accessible at https://github.com/hoaltay/CMA_PD.
